# Dr. Frank Henry Columbus Zornow

**Published:** 1918-01

**Authors:** 


**Dr. Frank Henry Columbus Zornow**, Pittsford, N. Y., New York Homoeopathic Medical College, New York City, 1917, age 24, an interne in the Rochester, N. Y., Homeopathic Hospital, died in that institution October 25, from diphtheria.

